# Mitochondria, cholesterol and cancer cell metabolism

**DOI:** 10.1186/s40169-016-0106-5

**Published:** 2016-07-25

**Authors:** Vicent Ribas, Carmen García-Ruiz, José C. Fernández-Checa

**Affiliations:** 1 Department of Cell Death and Proliferation, Institute of Biomedical Research of Barcelona (IIBB), Consejo Superior Investigaciones Cientificas (CSIC), Barcelona, Spain; 2 Liver Unit-Hospital Clínic, Centre Esther Koplowitz, IDIBAPS, CIBEREHD, Planta Cuarta, C/Rosselló 149, Barcelona, 08036 Spain; 3 Research Center for ALPD and Cirrhosis, Ckeck School of Medicine, University of Southern California, Los Angeles, CA USA

**Keywords:** Mitochondria, Cholesterol, Tumor metabolism, Warburg effect, Reactive oxygen species, Apoptosis

## Abstract

Given the role of mitochondria in oxygen consumption, metabolism and cell death regulation, alterations in mitochondrial function or dysregulation of cell death pathways contribute to the genesis and progression of cancer. Cancer cells exhibit an array of metabolic transformations induced by mutations leading to gain-of-function of oncogenes and loss-of-function of tumor suppressor genes that include increased glucose consumption, reduced mitochondrial respiration, increased reactive oxygen species generation and cell death resistance, all of which ensure cancer progression. Cholesterol metabolism is disturbed in cancer cells and supports uncontrolled cell growth. In particular, the accumulation of cholesterol in mitochondria emerges as a molecular component that orchestrates some of these metabolic alterations in cancer cells by impairing mitochondrial function. As a consequence, mitochondrial cholesterol loading in cancer cells may contribute, in part, to the Warburg effect stimulating aerobic glycolysis to meet the energetic demand of proliferating cells, while protecting cancer cells against mitochondrial apoptosis due to changes in mitochondrial membrane dynamics. Further understanding the complexity in the metabolic alterations of cancer cells, mediated largely through alterations in mitochondrial function, may pave the way to identify more efficient strategies for cancer treatment involving the use of small molecules targeting mitochondria, cholesterol homeostasis/trafficking and specific metabolic pathways.

## Introduction

Cancer cells exhibit critical metabolic transformations induced by mutations leading to gain-of-function of oncogenes and loss-of-function of tumor suppressor genes that result in cell deregulation associated with increased cellular stress. Hanahan and Weinberg identified the six conceptual hallmarks of human cancer: (1) self-sufficient growth signaling, (2) evasion of growth suppressors, (3) cell death resistance, (4) replicative immortalization, (5) angiogenesis and (6) invasion/metastasis [1]. Other common characteristics of cancer cells include enhanced anabolism, avoidance of immune destruction and altered autophagy [2, 3]. Of these characteristic features of cancer cells, mitochondria are directly involved in a number of them. Indeed, mitochondria are critical mediators of apoptosis and the source of reactive oxygen species (ROS) generation and energy production. Consequently, altered mitochondrial function of cancer cells underlies several phenotypes, including: (1) resistance to apoptosis; (2) increased biosynthetic anabolism to support uncontrolled growth and proliferation; (3) increased ROS generation that activates metastatic proteases, tumor-promoting inflammation, genetic instability and DNA mutagenesis; (4) decreased mitochondrial oxidative phosphorylation (OXPHOS), increased aerobic glycolysis and decrease of pH in the extracellular milieu. Furthermore, due to its role as a hub in several signaling pathways [4], mitochondria are central for key metabolic alterations of cancer cells, some of which will be described below.

Experimental evidence indicates that high cell proliferation [5, 6] and tumor growth [7, 8] are closely associated with enhanced cholesterol requirement. Some types of cancers, such as hepatocellular carcinoma (HCC), are dependent on cholesterol for growth [9], and observational studies show a protective association between the use of statins and the risk of developing liver cancer [10], although this trend has been also observed in other cancer types, such as prostate and gastrointestinal cancers [11]. In line with this, genome-scale metabolic models of hepatocellular carcinoma found that among 101 metabolites relevant to HCC development, 30 % of them are related to cholesterol biosynthesis [12]. This protective effect of statins has been attributed to the inhibition of the mevalonate pathway (see below), preventing the posttranslational modification of the oncogenes MYC, RAS and RHO [11, 13, 14]. Moreover, analyses of the Cancer Genome Atlas (TCGA) database revealed a correlation between increased activity of the cholesterol synthesis pathway and decreased survival in patients with sarcoma, acute myeloid leukemia and melanoma [15, 16], supporting the concept that cholesterol promotes carcinogenesis. In this regard, cholesterol trafficking to mitochondria has been reported in tumor cells [17, 18] and may account for the recognized mitochondrial dysfunction and contribute to chemotherapy and apoptosis resistance and metabolic reprogramming of cancer cells, which will be discussed in the following sections.

## Mitochondria in cell life and death

### Life-sustaining functions

Mitochondria are complex organelles, which differ from the often-held view of isolated, small rounded double-membrane structures. They constitute a dynamic network that continuously undergoes fusion and fission controlled by specific mechanisms [19], and have interactions with other cell structures such as cytoskeleton and endoplasmic reticulum (ER) [20, 21]. Mitochondria contain multiple copies of their own maternally-inherited mitochondrial DNA (mtDNA), with an epigenetic complexity not completely understood [22]. Mitochondrial DNA is a circular molecule of approximately 16.5 kilobases present from hundreds to thousands of copies per cell, which encodes 13 polypeptides of the OXPHOS and respiratory chain, as well as 2 ribosomal RNAs and 22 transfer RNAs necessary for translation of polypeptides inside mitochondria. Most mitochondrial proteins (approximately 1500) are encoded by nuclear DNA, translated in the cytosol and imported into the mitochondria through specific translocator complexes (TIM and TOM) of the mitochondrial inner (MIM) and outer membranes (MOM), respectively. In addition, a disulfide relay molecular device consisting of MIA40 and augmenter of liver regeneration (ALR) are responsible for the import of nuclear encoded sulfur Fe/S cluster proteins to the mitochondrial intermembrane space that are essential for mitochondrial function [23, 24]. Recent data have shown that ALR links mitochondrial function to HCC development [25, 26]. Indeed, mitochondrial proteome has significant cell-type differences, allowing mitochondria to serve in a highly adaptive fashion to the cellular specific functional requirements [27].

Mitochondria are the power plants of the cell, providing the energy for countless cellular functions through OXPHOS. OXPHOS is coordinated by a cascade of redox reactions organized in five protein complexes embedded in the MIM, known as the electron transport chain (ETC), which transfers electrons to oxygen [28, 29]. The fall in electron potential energy through the ETC is used to pump protons out of the mitochondrial matrix to the intermembrane space, generating an electrochemical gradient known as the mitochondrial transmembrane potential (Δψ_m_), which induces a proton motive force used by complex V to regenerate ATP from ADP. Moreover, many additional mitochondrial processes, especially those related to transport of solutes across the MIM [30] are dependent on the electrochemical driving force of the Δψ_m_. Additional metabolic pathways that are located within mitochondria comprise the tricarboxylic acid cycle (TCA or Krebs cycle), β-oxidation of fatty acids, steroidogenesis, metabolism of amino acids, formation of Fe/S clusters, heme biosynthesis as well as reactions involved in lipogenesis, gluconeogenesis, ketogenesis and ammonium detoxification (urea cycle) [31].

Physiologically under aerobic conditions, cells degrade glucose via glycolysis to pyruvate, which is imported into mitochondria. Pyruvate enters the TCA cycle in the form of acetyl-CoA that along with oxaloacetate generates citrate, in a reaction catalyzed by citrate synthase. Citrate is processed in the TCA cycle to generate reducing equivalents that feed the ETC and generate energy with the consumption of oxygen. However, in conditions where macromolecular biosynthesis is active, citrate may be exported to cytosol where is converted to acetyl-CoA by ATP citrate lyase (ACLY), which is used for lipogenesis. Besides their role in metabolism, mitochondria are involved in calcium homeostasis, innate immunity, integration of signaling pathways and autophagy [32, 33]. Moreover, in response to metabolic and genetic stress mitochondria and nucleus engage in bidirectional signaling pathways, which modulate cell function [4, 34, 35].

Electron transport through the ETC can leak the chain and react with oxygen to generate ROS [36, 37]. Complex I and complex III are the major sources of mitochondrial ROS generation [28], although other mitochondrial sites also contribute to ROS production, including complex II [38]. The existence of an efficient antioxidant defense system, of which mitochondrial glutathione (mGSH) is a central component, prevents or repairs oxidative damage generated during normal aerobic metabolism [39]. The primary ROS generated in mitochondria is superoxide [40], which is produced in the mitochondrial matrix and undergoes dismutation to hydrogen peroxide (H_2_O_2_) [40], a reaction catalyzed by mitochondrial superoxide dismutases (SOD2). Hydrogen peroxide is further inactivated by the mitochondrial glutathione peroxidase (mGSH/GPX) and peroxiredoxin/thioredoxin (Prx/Trx) antioxidant systems [41]. Both systems use the reducing equivalents of NADPH to regenerate the mitochondrial oxidized glutathione (mGSSG) and Trx back to the reduced forms. The Prx/Trx system is thought to be responsible for scavenging hydrogen peroxide at nanomolar concentrations, while mGSH/GPX system is important for buffering high ROS levels [42, 43]. However, both systems are mutually regulated, as selective depletion of mGSH results in decreased levels of Trx2 and Prx3 [44], highlighting the central role of mGSH in maintaining an adequate hydrogen peroxide homeostasis. Due to its more stable and diffusible nature, hydrogen peroxide acts as a second messenger because of its reactions with specific oxidation-prone protein cysteinyl residues [45], which confers properties to hydrogen peroxide as a mitochondrial signaling molecule [4]. In line with this, mitochondrial hydrogen peroxide bursts have self-sustained circadian oscillations, acting as a redox intracellular pacemaker [46].

### Death promoting pathways

Besides their fundamental role in energy generation, mitochondria also play a strategic role in the regulation of several forms of cell death, including apoptosis (both caspase-dependent and independent), necrosis and programmed necrosis [47]. The central mediators of apoptosis include a group of cysteine proteases named caspases, which become activated by a proteolytic processing cascade in response to pro-apoptotic signals. The series of events leading to apoptosis have been categorized in two modes, the extrinsic and intrinsic apoptotic pathways. The extrinsic pathway involves extracellular ligand binding to a transmembrane death receptor, such as TNF receptor or FAS receptor, followed by recruitment of cytosolic adaptor proteins and activation of an initiator caspase (usually caspase-8), which stimulates an effector caspase (such as caspase-3). Conversely, the intrinsic (or mitochondrial) pathway involves the destabilization of the MOM and the release of mitochondrial proteins that activate effector caspases. The BCL-2 family of proteins regulates this pathway with opposing pro-apoptotic effector functions (BAX, BAK), pro-apoptotic BH3-only proteins (BAD, BIM, BID, BIK, Noxa, PUMA, HRK, BMF) and anti-apoptotic functions (BCL-2, BCL-xL, MCL-1, A1, BCL-B, BCL-w) [48]. Activation of the intrinsic pathway of apoptosis by a number of stimuli and stresses, triggers the binding and activation of pro-apoptotic proteins BAX or BAK to the MOM leading to the MOM permeabilization (MOMP) without disruption of the inner membrane and the subsequent release of proteins from the mitochondrial intermembrane space (IMS), such as cytochrome *c* [49, 50]. Although active BAX or BAK are required to induce MOMP, the underlying mechanism is controversial [51]. While the model of pro-apoptotic activation or neutralization by anti-apoptotic members are still incompletely known, recent findings have shown that BCL-2 ovarian killer (BOK), which displays a high sequence similarity to BAX and BAK, engages the mitochondrial apoptotic pathway independently of BAK/BAX [52]. Although mitochondrial proteins are normally secured in the IMS the rupture of the physical barrier (MOM) constitutes a point-of-no-return in cell death [49, 50]. Pro-apoptotic BH3-only proteins act as stress sentinels that relay the diverse array of apoptotic signals via BAX/BAK activation to induce MOMP. In contrast, anti-apoptotic BCL-2-family proteins prevent MOMP and apoptosis by binding BH3-only proteins, preventing their interaction with BAX/BAK, or by binding activated BAX/BAK [53]. Pro- and anti-apoptotic BCL-2 protein interactions are mediated between BH-3 domains and the BH3 binding cleft in anti-apoptotic BCL-2 proteins.

Once released from the mitochondria into the cytosol through MOMP, cytochrome *c* binds to the adaptor molecule APAF-1, causing it to oligomerise and form a heptameric structure called apoptosome [54]. This complex recruits pro-caspase 9, which in turn, activates the executioner caspases-3 and -7, triggering the cascade of events that lead to controlled cell death and fragmentation. In addition to cytochrome *c*, other IMS proteins (Table 1) are also mobilized and released into the cytosol following MOMP where they promote or counteract caspase activation and hence cell death [55–60].

**Table 1:** IMS proteins related to apoptosis induction

IMS protein	MW (kDa)	Function	References
Cytochrome *c*	12	Apaf-1 binding and apoptosome initiation	[49, 54, 56]
SMAC/DIABLO	23	Neutralization of apoptosis inhibitor factors	[49, 56, 57]
OMI/HTRA2	37	Neutralization of apoptosis inhibitor factors	[49, 58]
AIF	62	DNA fragmentation	[56, 59]
ENDOG	28	DNA fragmentation	[55]
AK2, Adenylate Kinase 2	26	Initiation of AK2-FADD-caspase-10 complex	[60]

For the execution of mitochondrial apoptosis cytochrome *c* detaches from the MIM and dissociates from the phospholipid cardiolipin, which binds cytochrome *c* by an electrostatic bond [61]. Cardiolipin can be oxidized by ROS or by the cardiolipin–cytochrome *c* complex [62] resulting in oxidized cardiolipin, which exhibits lower affinity for cytochrome *c* than the reduced form, and therefore contributes to cytochrome *c* detachment from MIM and its release to cytosol. Since mitochondrial ROS are controlled by antioxidants [63, 64], mGSH arises as an important modulator of apoptotic cell death by indirectly controlling the redox state of cardiolipin [63, 65]. In addition, it has been described that oxidized cardiolipin modulates the biophysical properties of MOM to allow oligomerized BAX to insert and permeabilize the MOM [63, 65, 66].

Integrin-mediated attachment of normal cells to the extracellular matrix elicits anti-apoptotic and pro-survival signaling. The loss of cell–matrix interaction induces anoikis, a specific form of apoptosis [67]. Cell detachment leads to upregulation and activation of several BH3-only pro-apoptotic proteins (BID, BIM and BDF) that, in turn, activate BAX and BAK resulting in MOMP and the apoptotic cascade, resulting in cell death [68]. In addition to MOMP, the generation of mitochondrial ROS in cells undergoing anoikis is required for cell death, as antioxidants treatment suppressed anoikis [69, 70]. Normal cells detached from the matrix undergo dramatic global metabolic changes characterized by decreased mitochondrial respiration and SOD2 induction. Indeed, cells depleted of SOD2 are hypersensitive to cell death by anoikis [71], suggesting the importance of ROS generated in mitochondria in the execution of anoikis.

As opposed to apoptosis, necrosis is a morphologically distinct form of cell death responsible for irreversible tissue destruction due to bioenergetic failure and oxidative damage. Permeabilization of the MIM by the mitochondrial permeability transition (MPT) and secondary rupture of the MOM is a key event of necrosis. MPT is a regulated pore-forming protein complex whose molecular characterization remains elusive [72–74]. Of the MPT components, cyclophillin D is a key constituent, while the role of other putative components, such as voltage-dependent anion channel (VDAC), adenine nucleotide translocase (ANT) and translocator protein (TSPO, also called benzodiazepine receptor, PBR) is controversial [49, 75, 76]. Mitochondrial ROS regulate MPT by targeting specific cyclophillin D cysteine residues. Necrosis is characterized by mitochondrial swelling, loss of Δψ_m_, and impaired OXPHOS and ATP generation. The fundamental difference with respect to apoptosis is the rapid loss of cellular membrane potential due to energy depletion and ion pump/channel failure, leading to swelling and cytolysis. Concomitantly, water influx causes matrix swelling, rupture of MOM and release of apoptogenic proteins sequestered in IMS. These events, however, block apoptotic cell death due to energetic failure, ATP exhaustion and oxidative stress-mediated caspase inactivation. Moreover, TNFα has been recently shown to induce a caspase-independent form of programmed cell death, named programmed necrosis or necroptosis [77, 78], involving receptor-interacting serine/threonine-protein kinase 1 (RIPK1) and RIPK3 kinases, which interact with the pseudokinase mixed lineage kinase domain-like protein (MLKL). The execution of necroptosis requires mitochondrial ROS generation, which is dependent of MPT and involves cyclophyllin D but it is independent of BAX or BAK [79].

## Cholesterol homeostasis and mitochondrial trafficking

### Cholesterol synthesis and deregulation in cancer cells

Cholesterol is an essential component of membrane bilayers that plays a key role in their integrity and function. While intake of cholesterol from the diet ends up in different cell membranes, the predominant mechanism that provides the cholesterol needed for cellular functions is its de novo synthesis from acetyl-CoA in the so-called mevalonate pathway, which generates not only cholesterol but also non-sterol components, such as dolichol, ubiquinol and isoprenoids. The hydroxymethylglutaryl-CoA reductase (HMGCoAR) catalyzes the reduction of HMG CoA to mevalonate, the rate-limiting step in the synthesis of cholesterol [80]. Mevalonate is phosphorylated to pyrophosphomevalonate, which is then converted to isopentenyl pyrophosphate (IPP). IPP can be reversibly transformed to dimethylallylpyrophosphate (DMAPP), which can combine with IPP to generate the 10-carbon isoprenoid geranyl pyrophosphate (GPP). The secuential addition of 1 or 2 more IPP units to GPP generates farnesyl pyrophosphate (FPP) and geranylgeranyl pyrophosphate (GGPP), respectively. Isoprenoids generation in the mevalonate pathway is an essential mechanism of posttranslational modification of proteins and these lipid moieties anchor target proteins to cell membranes. FPP is used to prenylate proteins of the Ras family, while GGPP prenylates those of the Rho family [81]. In addition, FPP can be converted into squalene by squalene synthase (SS), which catalyzes the first step in the committed pathway for cholesterol synthesis. Statins, whose chemical structure is similar to that of HMGCoA, compete with and inhibit HMGCoAR, preventing the formation of mevalonate and its downstream product IPP. Therefore, the therapeutic effects of statins can extend beyond cholesterol inhibition and impact in the regulation of a number of proteins due to the blockade of isoprenoids (FPP and GGPP) generation. In contrast to statins, the inhibition of SS results in selective cholesterol downregulation without exerting a major effect in the isoprenylation of proteins [82].

As HMGCoAR is the regulatory enzyme in the mevalonate pathway its feedback and transcriptional control impact in cholesterol and isoprenoids regulation. One mechanism for feedback control involves the rapid degradation of HMGCoAR mediated by ER resident proteins, Insigs. Accumulation of sterols in the ER membrane triggers binding of the membrane domain of HMGCoAR to a subset of Insigs, which carry a membrane-anchored ubiquitin ligase called GP78 which ubiquitinates HMGCoAR, marking it for proteasomal degradation [83]. HMGCoAR is regulated at the transcriptional level by the transcription factor SREBP-2, which resides in the ER is an inactive form. When sterols levels are low, SREBP-2 is transported from the ER to the Golgi to undergo a proteolytic processing by specific proteases, resulting in the mature form of SREBP-2, which translocates to the nuclei to induce HMGCoAR as well as other targets involved in the regulation of cholesterol homeostasis, including the LDL receptor.

As cholesterol synthesis requires oxygen, which is used for the biotransformation of squalene to cholesterol, an additional mechanism that regulates cholesterol synthesis is oxygen availability. Indeed, the bulk for the oxygen requirement centers on the sequential transformation of lanosterol to cholesterol, involving several redox reactions. Moreover, hypoxia has been shown to stimulate HMGCoAR degradation through both accumulation of lanosterol and Insigs induction [84]. In contrast to these physiological features, cholesterol synthesis and regulation are altered at several levels in cancer cells to meet the unrestricted growth needs [84–87]. Indeed, tumor cells exhibit increased cholesterol levels compared to surrounding cells; moreover, cancer tissues display increased upregulation of HMGCoAR, loss of feedback inhibition, decreased expression of cholesterol exporter ATP binding cassette transporter A1 (ABCA1) and increased extracellular cholesterol uptake via LDL receptor [87]. Hence, as briefly described below (“Strategies targeting the mevalonate pathway and cholesterol synthesis in cancer” section), targeting the mevalonate pathway may be of potential relevance in cancer therapy.

### Mitochondrial cholesterol trafficking in cancer

Mitochondria are cholesterol-poor organelles compared to other cell bilayers (e.g. plasma membrane). Nevertheless, the limited availability of cholesterol in the MIM plays an important physiological role, including the synthesis of bile acids in hepatocytes or steroid hormones in specialized tissues through the metabolism of mitochondrial cholesterol by CYP27A or CYP11A1, respectively. In pathological conditions, however, the accumulation of cholesterol in mitochondria alters membrane organization and the coexistence of lipid-disordered and lipid-ordered phases, which regulates membrane permeability and function of resident proteins [88]. Of relevance, increased mitochondrial cholesterol levels have been described in solid tumors. For instance, mitochondrial cholesterol levels of tumors from Buffalo rats bearing transplanted Morris hepatomas are two to fivefold higher than the content found in mitochondria prepared from host liver, and correlated with the degree of tumor growth and malignancy [89, 90]. As mitochondrial cholesterol in cancer cells contribute to the alterations in mitochondrial function and properties, understanding the mechanisms governing the trafficking of cholesterol to mitochondria may be of relevance in cancer cell biology. In this regard, given its lipophilic properties and water insolubility, non-vesicular transport by specific carriers stands as the major mechanism of cholesterol transport between organelles. In particular, mitochondrial cholesterol transport is preferentially regulated by the steroidogenic acute regulatory domain 1 (StARD1), the founding member of a family of lipid transporting proteins that contain StAR-related lipid transfer (START) domains [91]. StARD1 is a MOM protein, which was first described and best characterized in steroidogenic cells, where it plays an essential role in cholesterol transfer to MIM for metabolism by CYP11A1 to generate pregnenolone. Despite similar properties with StARD1, other StART members cannot replace StARD1, as germline StARD1 deficiency is lethal due to adrenocortical lipoid hyperplasia [92]. Moreover, targeted mutations in MLN64 (StARD3), another START member with wide tissue distribution, impair steroidogenesis while causing minor alteration in cholesterol metabolism [93]. Furthermore, analyses of the TCGA database further support a role for StARD1 and MLN64 and subsequent mitochondrial cholesterol enrichment in cancer development. Although MLN64 is an endosomal protein, it participates in the egress of cholesterol from endosomes to mitochondria [94], suggesting that MLN64 and StARD1 work in concert to ensure the trafficking of cholesterol to MIM. Increased StARD1 expression and mitochondrial cholesterol loading are causally linked as StARD1 silencing decrease mitochondrial cholesterol levels in hepatocellular carcinoma [17]. Moreover, decreased ABCA1 activity has been reported in colorectal cancer cells either through loss-of-function or gene downregulation and ABCA1 downregulation promoted cancer cell survival by increased mitochondrial cholesterol accumulation [95]. Thus, these findings indicate that the trafficking and accumulation of cholesterol in mitochondria is a characteristic feature of many types of cancer and its role in carcinogenesis may be related to the regulation of cell death and chemotherapy sensitization, which will be described below.

## Role of mitochondria and cholesterol in altered cancer cell metabolism

The oncogenic transformation of cancer cells requires energy metabolism reprogramming in order to support unrestrained growth. The dependence on aerobic glycolysis despite normal oxygen tension constitutes one of the key metabolic alterations in cancer cells. This event was first described by Otto Warburg in 1930 and has ben coined since then as the *Warburg Effect* [96–98]. Although the glycolytic phenotype in cancer cells was proposed to be due to defective mitochondrial OXPHOS, many cancer cells exhibit competent OXPHOS activity capable to generate ATP [99]. The dependence on glycolysis is characteristic of many tumors and is widely exploited for clinical tumor imaging using positron emission tomography (PET) with a radiolabeled analog of glucose (^18^F-fluorodeoxyglucose) [100]. Elevated aerobic glycolysis in cancer cells serves many purposes, ensuring ATP generation without reliance on oxygen availability. Moreover, aerobic glycolysis generates bicarbonic and lactic acids, which are released to the extracellular milieu, favoring tumor invasion, angiogenesis and immunosurveillance suppression [101]. Glucose can be diverted to the pentose phosphate pathway to generate nucleotides and NADPH to fuel antioxidant defenses and biosynthetic reactions. Finally cancer cells use intermediates of glycolytic pathway for biosynthesis of de novo nucleic acids, lipids and amino acids to support their unrestrained growth and proliferation [97, 102, 103]. In line with these changes, a Warburg-like metabolism has been described in many rapidly proliferating embryonic tissues, supporting the biosynthetic programs of aerobic glycolysis in active proliferating cells [104, 105]. Given that many tumor types rely on oxidative metabolism, glucose flux is not necessarily coupled to oxidative glucose metabolism. Oxygen consumption in many cancer cells is used for mitochondrial oxidation of alternate fuels, such as glutamine [106], suggesting that the fate of glucose for mitochondrial oxidation in cancer cells is probably even lower. Cancer cells undergo a number of metabolic alterations, including the depression of oxidative mitochondrial OXPHOS and TCA cycle, which are used for anabolic reactions [107, 108]. Moreover, several transcriptional and posttranslational mechanisms have been proposed to contribute to the metabolic reprogramming and dependence on the Warburg effect in cancer cells, involving activation of oncogenes and inactivation of tumor suppressor genes. In this regard, activated oncogenes such as KRAS and MYC along with mutated tumor suppressors such as TP53 can extensively reprogram cell metabolism resulting in diversion of carbon skeletons to fuel anabolic reactions for biomass synthesis instead of being completely oxidized through mitochondrial respiration.

### MYC in tumor metabolism reprogramming

MYC is an oncogene that plays a role in cell cycle progression, apoptosis and cellular transformation. In addition, MYC is important for the increased transcription of metabolic enzymes required for anabolism in cancer and fast-growing cells, regulating the conversion of glucose to pyruvate through the activation of important glycolytic genes and glucose transporters, while blocking the entry of pyruvate into the TCA cycle via pyruvate dehydrogenase kinase (PDK1). Interestingly, MYC promotes the metabolic adaptation of tumor cells [109] by activating genes important for mitochondrial biogenesis and function [110, 111]. Moreover, the AMPK-related protein kinase 5 (ARK5), which is involved in maintenance of mitochondrial integrity and bioenergetic homeostasis, was identified as a MYC target [112]. This dual role of MYC as a driver of Warburg effect and a promoter of mitochondrial biogenesis underlies the dependence of cancer cells on glutamine oxidation, an essential event for cell survival under conditions with low glucose and oxygen [113]. Moreover, MYC upregulates the glutamine transporters SLC5A1 and SLC7A1, which contribute to glutamine uptake in cancer cells. As MYC induces the flux of 3-phosphoglycerate from glycolysis to the synthesis of serine and glycine needed for nucleotide biosynthesis, MYC coordinates the synthesis of nucleotides with glutamine metabolism [114]. Indeed, the rate of glutaminolysis is greater compared to the rate of glycolysis in cells with high MYC expression and are more dependent on mitochondrial oxidative metabolism than cells with low MYC levels.

Tumors are metabolically heterogeneous, exhibiting complex metabolic profiles [115], including the dependence on aerobic glycolysis and reliance on OXPHOS [116–119]. For instance, while cancer stem cells are quiescent and exhibit high OXPHOS reliance, they may coexist with other highly cycling cancer cells that rely on glycolysis. The dependence of these cancer stem cells on mitochondrial OXPHOS prompted the use of mitochondrial OXPHOS inhibitors to selectively target these cells to prevent tumor relapse after cytotoxic treatment [120, 121]. It has been described that in pancreatic tumors MYC acts as a switch between the OXPHOS-dependent metabolism of cancer stem cells towards the highly glycolytic differentiated progeny, creating a gradient of heterogeneous oxidative/glycolytic population inside the tumor. Moreover, MYC acts as a direct transcriptional inhibitor of peroxisome proliferator-activated receptor α (PGC1-α) suppressing mitochondrial respiration while activating glycolytic programs [119]. This heterogeneity defines a scenario where therapies targeting specifically highly respiratory or highly glycolytic tumor cells may not be completely effective.

### TP53 and tumor metabolism

Reduced expression of the tumor suppressor protein TP53 can also impact metabolic reprogramming in cancer cells. Defects in P53 function lead to impaired transactivation of SCO2, a mitochondrial protein required for the correct assembly of the cytochrome *c* oxidase in the ETC and of TIGAR, an isoform of 6-phospho-fructo-2-kinase, whose expression exerts a tumor suppressor function by inhibiting glycolytic flux [122, 123]. Moreover, TP53 activates transcription of glutaminase 2 (GLS2) to promote glutaminolysis to fuel the TCA cycle and facilitate fatty acid oxidation as an alternative source [124]. Collectively, TP53, in addition to its role in orchestrating cell cycle arrest and apoptosis, counteracts the Warburg effect by favoring OXPHOS and minimizing glycolytic metabolism, and therefore its loss-of-function is a requirement for the aerobic glycolysis in most carcinogenic processes.

### Hypoxia-inducible factor (HIF1α)

**Fig. 1:**
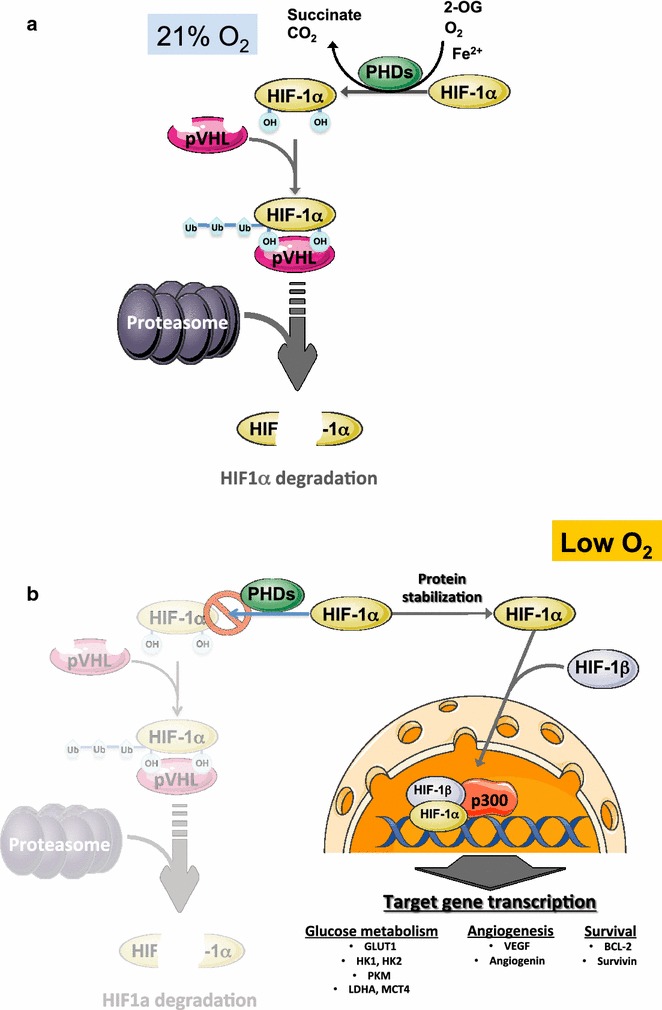
Regulation of HIF1α transcriptional activity. **a** In normoxia, PHD hydroxylates HIF1α in several proline and asparagine residues, with 2-OG, ascorbate and Fe^3+^, acting as cofactors for the reaction. Hydroxyted HIF1α binds with high affinity to the E3 ubiquitin-ligase pVHL and HIF1α becomes ubiquitinated, marking it for proteasomal degradation. Through this mechanism, HIF1α is kept at very low concentrations and transcriptionally inactive. **b** In low O_2_ conditions, the activity of PHD is inhibited due to lack of oxygen, resulted in no hydroxylation nor ubiquitination of HIF1α. These events lead to the HIF1α protein stabilization which can translocate to the nucleus where it forms a complex with HIF1β and recruits CBP/p300 to the promoter of HIF1α target genes, activating the transcription of a vast array of genes responsible of glycolysis, angiogenesis and cell death resistance which are involved in tumor progression

Hypoxia is an inherent feature of solid tumor development that arises due to the disorganized structure and architecture of tumor vasculature resulting in irregular and inefficient oxygen delivery. Hypoxia is considered a negative prognostic factor for response to treatment and survival of cancer patients [125, 126]. Hypoxia-inducible factor (HIF) is a key transcription factor activated mainly by hypoxia due to the dependence of HIF-proly hydroxylases (PHD) on oxygen (see below). In addition to hypoxia HIF is also regulated by oxidative stress, inflammation and metabolic stress [127]. HIF1 comprises a stable β subunit (HIF-1β/Arnt) and a labile α subunit (HIF1α) encompassing three family members, HIF1α, HFI2α and HIF3α (Fig. 1). In normoxia HIF1α is rapidly degraded due to the sequential action of oxygen-dependent PHD and the Von Hippel-Landau E3-ubiquitin ligase (pVHL). PHDs primarily function as oxygen sensors so that in normoxia PHDs become activated to hydroxylate HIF1α on two highly conserved proline residues. Hydroxylated HIF1α is then recognized and ubiquitinated by the pVHL, marking HIF1α for proteasomal degradation (Fig. 1a). In low oxygen conditions, PHDs are inactivated and therefore HIF1α is stabilized, translocate to the nucleus where heterodimerize with HIF1β/Arnt to form a complex that activates hundreds of genes involved in energy metabolism, autophagy and angiogenesis [128] (Fig. 1b). Activation of HIF1α promotes the conversion of glucose to pyruvate and lactate by upregulating the transcription of glucose transporters (GLUT1), hexokinases (HK1 and HK2), lactate dehydrogenase A (LDHA) as well as the lactate-extruding monocarboxylate transporter 4 (MCT4) [129], supporting the shift to aerobic glycolysis. Activated HIF1α increase the transcription of the PDK1, which inhibits PDH, decreasing the conversion of pyruvate to acetyl-CoA, which compromises OXPHOS, therefore linking low oxygen conditions to the depression of mitochondrial function. Moreover, HIF-1 activates transcription of the cytochrome *c* oxidase subunit 4-2 (COX4-2) and the LON mitochondrial protease, which degrades COX4-1 subunit and allows its substitution by the less efficient COX4-2 subunit [130]. In a scenario with inhibited mitochondrial OXPHOS by genetically downregulating the master regulator of mitochondrial biogenesis PGC1α, ROS-mediated HIF1α stabilization is able to rescue cell bioenergetics by activating transcription of glycolytic genes and glycolysis, allowing cancer cells to escape from metabolic stress [131]. In addition, HIF1 α induces the expression of BCL-2/adenovirus E1B 19-kDa interacting protein 3 (BNIP3) and BNIP3-like (BNIP3L), which trigger mitochondrial autophagy, thereby decreasing the oxidative metabolism of both fatty acids and glucose [132]. Therefore, HIF1α not only counteracts the MYC-mediated suppression of mitochondrial biogenesis by reducing mitochondrial mass and function, but also cooperates with MYC to promote aerobic glycolysis by induction of HK2 and PDK1 [133]. There are three PHDs known in mammals, encoded by three genes (PHD1, PHD2 and PHD3) [134]. Although, PHDs are thought to act as true oxygen sensors due to their requirement of oxygen for hydroxylation of HIF1, they are also dependent on iron (Fe^2+^), ascorbate and on the TCA intermediate 2-oxoglutarate (2-OG) as cofactors. Conversely, it has been reported that several TCA intermediates, such as fumarate and succinate competitively inhibit all three PHDs, while citrate and oxaloacetate inhibit factor inhibiting HIF1 (FIH), an asparaginyl hydroxylase which is able to block the transcriptional activity of HIF1α by catalyzing the hydroxylation of an asparagine residue of HIF1α [135]. These effects have important implications as succinate dehydrogenase (SDH) inactivation and isocitrate dehydrogenase (IDH) neomorphic gain-of-function leading to accumulation of succinate and 2-hydroxyglutarate, respectively, contribute to HIF1α stabilization and cancer promotion [136].

### Role of ROS in cancer cell biology

The impact of ROS in cancer research is controversial due to their dual role in promoting tumor growth, angiogenesis and metastasis or supression of tumor development, depending on the context and on the type of species generated [137–140]. ROS are highly reactive molecules with the potential to target and oxidize proteins, lipids and DNA, which derive from different sources and mechanisms (Table 2) and from environmental events, such as ultraviolet or ionizing radiation [39, 139, 141].

**Table 2:** Cellular sources of ROS

Source	Cellular compartment	Primary radical generated
Complex I_F_	Mitochondria	$\rm{O_{2^-}}$
Complex I_Q_	Mitochondria	$\rm{O_{2^-}}$
Complex II_F_	Mitochondria	$\rm{O_{2^-}}$
Complex II_Q0_	Mitochondria	$\rm{O_{2^-}}$
mGPDH	Mitochondria	$\rm{O_{2^-}}$
ETFQOR	Mitochondria	$\rm{O_{2^-}}$
PDH	Mitochondria	$\rm{O_{2^-}}$
OGDH	Mitochondria	$\rm{O_{2^-}}$
BCKDH	Mitochondria	$\rm{O_{2^-}}$
P66shc	Mitochondria, cytoplasm	H_2_O_2_
NOS	Cytoplasm	NO
NOX family	Cytoplasm, cell membrane	$\rm{O_{2^-}}$
Xantine oxidase	Cytoplasm, peroxisome	H_2_O_2_
Cytochrome p450 family	Endoplasmic reticulum	$\rm{O_{2^-}}$ H_2_O_2_

*CI*_*F*_ complex I flavin site, *CI*_*Q*_ complex I ubiquinone site, *CII*_*F*_ complex II flavin site and *CIII*_*Q0*_ complex III_Qo_ are sites of the mitochondrial ETC, *mGPDH* Mitochondrial glycerol 3-phosphate dehydrogenase, *ETFQOR* electron-trasferring flavoprotein ubiquinone oxidoreductase, *PDH* pyruvate dehydrogenase, *OGDH* 2-oxoglutarate dehydrogenase and *BCKDH* branched-chain 2-oxoacid dehydrogenase are mitochondrial enzymes capable of generate ROS. Upon stress signaling, cytosolic p66Shc translocates to mitochondria to directly stimulate hydrogen peroxide generation. Nitric oxide synthase (NOS) produces NO^.^by facilitating the conversion of l-arginine to l-citruline. NADPH oxidase family of enzymes (NOX) transfer electrons from NADPH to O_2_ to produce O_2_^−^. Other cellular enzymes incuding xanthine oxidase and cytochrome p450 families also participate in ROS generation in normal biological reactions and in chemicals or xenobiotics detoxification reactions

ROS-induced damage on DNA can lead to enhanced mutation rates, driving the transformation of normal cells into a tumorigenic phenotype. In line with this association, moderate intake of antioxidants have shown to reduce the risk of cancer development and slow cancer progression [142–145], leading to the concept that antioxidants can prevent ROS-induced damage and therefore cancer incidence. Moreover, high ROS production in cancer cells can stabilize survival factors such as HIF1α, which drive tumor initiation and progression [146]. Solid tumor formation, in turn, contributes to hypoxia development due to the disorganized vasculature, and the limited oxygen supply in solid tumors stimulates mitochondrial ROS generation and HIF1α stabilization [147, 148]. HIF1α in turn activates ROS generation, establishing a feed-forward loop where HIF1α supports its stability to promote cancer cell survival and malignant progression [141]. However, transformed cells adapt to this oxidative environment by turning on strategies that control the generation of ROS to ensure their role in proliferation signaling, while containing the damaging effects of ROS overproduction. An important strategy in this regard is the modulation of mitochondrial ROS generation, which is downregulated in cancer cells by shifting to aerobic glycolysis. This scenario suggests that reducing mitochondrial oxidation not only promotes survival of cancer cells but also increases anabolic metabolism. On the other hand, the pro-apoptotic activity of mitochondrial inhibitors are reversed by antioxidants [121, 149], lending further support for the association of ROS with tumor prevention [141]. Conversely, large-scale multicenter clinical trials of antioxidant supplementation showed a significant increase in cancer incidence [150–154]. Quite intriguingly recent preclinical studies confirmed the pro-tumorigenic and pro-metastatic effects of antioxidant supplementation such as *N*-acetyl-l-cysteine (NAC), a GSH precursor [155, 156], thus highlighting the relevance of antioxidants in the protection of cancer cells against oxidative damage. Therefore, antioxidant supplementation can promote the growth of tumors by rescuing the viability of cells under high oxidative stress.

A key survival strategy of cancer cells is the upregulation of antioxidant systems to detoxify the production of ROS. One central factor associated to the resistance of cancer cells is the transcription factor NF-E2-related factor (NRF2). NRF2 is a master regulator of the antioxidant response and xenobiotic metabolism through the regulation of a wide range of antioxidant and detoxification genes [157]. NRF2 is sequestered in the cytoplasm by the Kelch-like ECH-associated protein 1 (KEAP1), which acts as a NRF2 repressor and plays a pivotal role in the regulation of the NRF2 pathway. KEAP1 binds and promotes NRF2 degradation through the ubiquitin–proteasome pathway. Under oxidative stress or through particular chemical inducers, cysteine residues of KEAP1 are modified and the resulting conformational change leads to the release of NRF2, which is stabilized and translocated to the nucleus, to induce the transcription of a large number of genes [158]. In this regard, NRF2 activators, such as curcumin, butylated hydroxyanisole (BHA) or the synthetic oleane triterpenoids (CDDO), have preventive properties against carcinogenesis [157]. However, given the dual role of ROS on cancer genesis and development, NRF2 activation also provides protection to cancer cells. Therefore, NRF2 is constitutively elevated in many types of cancer cells [159–162] and this increase is associated with a poor prognosis in cancer patients [163–165]. A variety of molecular mechanisms contribute to the constitutive expression and/or stabilization of NRF2 in cancer cells. Loss-of-function by somatic mutations or epigenetic silencing of KEAP1 impairs its binding to NRF2 and abrogates its repressive effect [159, 166]. The autophagy protein P62, also named sequestrosome 1, binds and sequesters KEAP1 in autophagosomes, leading to the autophagy-dependent KEAP1 degradation, resulting in increased NRF2 stability and activation of target genes [167–170]. Overexpression of P62 or increased P62 levels due to defects in autophagy leads to persistent activation of NRF2 [171, 172], contributing to carcinogenesis [173]. In addition, activation of oncogenes such as *K*-RAS, BRAF and MYC stimulates the transcription of NRF2 [174]. There is substantial evidence that impaired TCA cycle activates NRF2 [175], in a similar fashion as described for HIF1α. In this case, fumarate accumulation can form adducts with KEAP1 on its cysteine residues and provoke NRF2 activation. Physiological fumarate levels are low due to the activity of fumarate hydratase (FH). However, in cancer cells with loss-of-function of FH, high levels of fumarate are associated with sustained NRF2 activation [176, 177]. Nonetheless, activation of NRF2 transcriptional activity leads to the upregulation of antioxidants and detoxifying enzymes that promote not only the survival of cancer cells but also mediate chemoresistance [178, 179]. Besides these important roles of NRF2 on detoxification, it has also been shown that NRF2 can contribute to other aspects of cancer survival such as the counteraction of cell death by BCL-2 overexpression [180] and altered metabolism by redirecting glucose and glutamine to the production of ribose-5-phosphate for nucleotide synthesis and to the regeneration of NADPH through the activation of the pentose-phosphate pathway [181].

Besides the role of ROS scavenging in cancer progression, this event is also important for cancer metastasis. Hence, it can be postulated that the supplementation of antioxidants would provide an additional advantage for cancer cells to spread to distant sites by counteracting their sensitivity to anoikis and oxidative stress. For instance, metastatic cells undergo reversible metabolic changes that allow them to counteract oxidative stress [156, 181]. Indeed, it has been recently shown that increased GSH synthesis mediates the metastatic colonization of colorectal cancer cells to the liver [182]. Conversely, other reports showed that inhibition of mitochondrial oxidative stress prevents metastasis [183, 184] and this apparent paradox might be explained by the different targets of antioxidants and their effect in different types of cancer cells [184–186]. Therefore, current antioxidant strategies are not clinically effective in cancer therapies, illustrating our limited understanding on the complex role of ROS in tumor initiation, progression and metastasis, which needs to be fully characterized to identify new and more effective therapeutic venues.

### Mitochondrial cholesterol in HIF1α regulation

As mentioned above, HIF-1 is the main transcription factor regulating the cellular response to hypoxia and its stabilization is known to promote cell survival and tumor progression. While HIF-1 stabilization is mainly determined through oxygen sensing by PHD and iron availability, PHD activity is also dependent on the cytosolic levels of 2-OG. Indeed, 2-OG emerges as a potential inhibitor of angiogenesis and cellular transformation by promoting the degradation of HIF1 [187, 188].

**Fig. 2:**
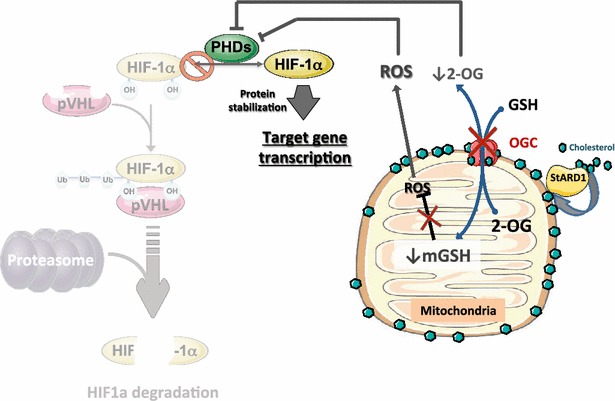
Mitochondrial cholesterol-mediated HIF1α stabilization. Mitochondrial cholesterol loading mediated by StARD1 decreases mitochondrial membrane fluidity which leads to an impaired activity of the OGC, which exchanges mitochondrial 2-OG by cytosolic GSH. Cytosolic 2-OG depletion may promote HIF1α stabilization by the impairment of PHD due to their requirement of 2-OG as a cofactor for HIF1α hydroxylation and subsequent degradation. Moreover, 2-OGC inhibition results in mGSH depletion, which in turn, limits the detoxification of ROS, in particular, H_2_O_2_. The subsequent increase in ROS and oxidative stress impact negatively on PHD, resulting in HIF1α stabilization

HIF-1α activation contributes to the metabolic reprogramming of cancer cells by impairing mitochondrial phosphorylation and the subsequent stimulation of aerobic glycolysis. Although the physiological levels of mitochondrial cholesterol are low, mitochondrial cholesterol accumulation impairs mitochondrial function and the activity of the mitochondrial 2-oxoglutarate carrier (2-OGC), which exchanges cytosolic GSH by matrix 2-OG. As StARD1 promotes mitochondrial cholesterol accumulation in the inner membrane, StARD1 induction thus contributes to the impairment of OGC carrier, resulting in the depletion of 2-OG in the cytosol and GSH in the mitochondrial matrix (Fig. 2). As mentioned above, mGSH is a key mitochondrial antioxidant that controls hydrogen peroxide production [39, 189, 190]. Moreover, mitochondrial ROS generation has been shown to promote HIF-1 α stabilization [147, 191]. Thus, it is conceivable that StARD1 induction and the subsequent accumulation of cholesterol in mitochondria result in the depletion of cytosolic 2-OG, impairing PHD activation and subsequent HIF-1α stabilization. Therefore, mitochondrial cholesterol loading may have an important role in cancer cell survival by a dual effect through impairment in mitochondrial function and dynamics, while promoting HIF1α stabilization via depletion of cytosolic 2-OG levels and generation of mitochondrial ROS. As a result, mitochondrial cholesterol loading in cancer cells acts as an additional mechanism governing angiogenesis and novel vessel growth via HIF1α stabilization, although this molecular link deserves to be further tested and it is currently under investigation. Finally, histone lysine demethylases have been recognized as important players in cancer cell biology by removing methyl moieties from DNA and aberrant expression of these chromatin modifying enzymes is implicated in the course of tumor initiation and progression [192]. Like PHD, histone methyl demethylases are also dependent on iron and 2-OG, and therefore mitochondrial cholesterol loading may further modulate cancer progression by the regulation of histone lysine demethylases via limitation of cytosolic 2-OG levels, which deserves further investigation.

## Role of mitochondria and cholesterol in cancer cell death and chemotherapy resistance

### Mitochondria and cell death resistance

Cancer cells have evolved multiple mechanisms to disable programmed cell death to support their survival and proliferation. Given that mitochondria are key players in several pathways of programmed cell death (see above) many strategic battles regulating cell death resistance take place in mitochondria [49]. The most prominent example of this is the overexpression of pro-survival BCL-2 proteins, a common feature in diverse cancers. The gene encoding BCL-2 was first identified in a chromosomal translocation that resulted in constitutively high levels of BCL-2 in neoplastic B cells [193, 194]. Different mechanisms such as genomic copy number amplification, oncogenic transcriptional upregulation or downregulation of microRNA repressors or stabilization of BCL-2 family members contribute to the maintenance of high levels of Bcl-2 [195, 196]. On the other hand, due to genomic deletion or promoter methylation leading to transcriptional silencing, loss-of-function of several pro-apoptotic proteins such as BAK, BAX and BH3-only family members have been observed in a variety of cancer types. Although BAX and BAK can play redundant roles, recent experimental data argues that in the context of activation of BH3-only protein or anti-apoptotic BCL-2 there is a strict dependence of either BAX or BAK [197, 198]. Although cancer cells are generally resistant to apoptosis, certain stress conditions, such as hypoxia and low nutrient availability, lower the threshold for apoptosis susceptibility. Cancer cells often exhibit higher levels of pro-apoptotic BH3-only protein, which is accompanied by higher anti-apoptotic BCL-2 proteins to antagonize apoptosis. This state has been termed as cancer cells “primed for death” [199] and this dependence on anti-apoptotic BCL-2 proteins can be exploited to design more effective pro-apoptotic therapeutic strategies [200].

Loss–of-function of TP53 is found in more than 50 % of human cancers. In addition to the above-mentioned roles of its inactivation in cancer cell metabolism, TP53 is central in the orchestration of cell death pathways upon cellular stress such as DNA damage by stimulating the transcription of pro-apoptotic proteins (PUMA, BAX), autophagy and cell-cycle arrest. TP53 exerts a vast array of extranuclear functions and therefore the cytoplasmic pool of TP53 cooperates with its nuclear counterpart to activate programmed cell death in response to certain cellular stresses. TP53 is involved in various forms of cell death such as apoptosis, necrosis and necroptosis and is able to mediate both MOMP and MPT opening in response to death stimuli. After stress induced TP53 activation, a small fraction translocates to MOM, resulting in the activation of the intrinsic apoptotic pathway [201]. TP53-mediated MOMP is related to the ability to bind and inactivate anti-apoptotic BCL-2 and BCL-xL, and to transcriptionally induce the expression and activation of pro-apoptotic proteins by direct binding [202]. Moreover, TP53 regulates MPT openings of necrosis/necroptosis via cyclophillin D and dynamin-related protein 1 (DRP1) [203, 204] in response to specific cell death triggers, such as TNFα or oxidative stress. In addition, TP53 inhibits autophagy [205], resulting in impaired mitophagy, contributing to the reduced threshold for cell death. Given these protective roles against specific alterations in cell cycle and cell death resistance, many cancer-associated TP53 mutations have been identified. Although most of TP53 mutations has been described as loss-of-function, it has been proposed that some TP53 mutations may have oncogenic capabilities [206].

Although MOMP is considered a point of no return for apoptosis, cancer cells are able to inhibit caspases ensuring survival in certain conditions. This mechanism described in some post-mitotic cells, such as neurons and certain cancer cells, allows the recovery of cancer cells provided that MOMP-inducing stimuli are removed [207–209]. Caspases can be directly inhibited by XIAP or by the neutralization of its inhibitors [200]. In addition, cytochrome *c* released through MOMP can be targeted for proteasomal degradation thereby avoiding the assembly of the apoptosome [209]. Besides caspase inhibition, survival after MOMP requires a pool of intact mitochondria in which MOMP has not been triggered [210]. The selective maintenance of cells with intact mitochondria may contribute to carcinogenesis and cancer relapse after cytotoxic therapies due to the increased susceptibility to oncogenic transformation [211]. Moreover, limited mitochondrial permeabilization induced by sub-lethal apoptosis triggers can promote DNA damage, genomic instability and ultimately carcinogenesis [212, 213]. This mechanism would have two important implications for cancer progression. First, low-level limited apoptosis can drive mutagenesis in surviving cancer cells, serving as a driving force towards malignancy. Second, sub-lethal apoptotic anticancer therapies can increase the tumorigenic potential of surviving cancer cells by promoting new mutations that favor relapse and chemotherapy resistance.

### Mitochondrial cholesterol in cell death and chemotherapy resistance

As mentioned above, cholesterol trafficking to mitochondria has been reported in tumor cells, including mitochondria from HCC due to overexpression of StARD1 [17]. Mitochondrial cholesterol loading in cancer cells may account for the recognized mitochondrial dysfunction and resistance to BAX-mediated cell death induced by chemotherapeutic agents that target mitochondria to elicit MOMP. In line with this, treatments that resulted in mitochondrial cholesterol loading in tumor cells impaired stress-induced apoptosis [17, 18], while StARD1 knockdown or treatments that resulted in downregulation of cholesterol loading sensitized HCC cells to chemotherapy [17]. Isolated mitochondria from HCC with increased cholesterol levels have been reported to be resistant to MOMP and release of cytochrome *c* or smac/DIABLO in response to various stimuli, such as MPT triggers and active BAX. In agreement with these findings, HeLa cells treated with the amphiphilic amine U18666, which perturbs intracellular cholesterol trafficking and stimulates mitochondrial cholesterol accumulation, impairs MOMP and the release of cytochrome c in response to BAX [18]. Furthermore, ABCA1 downregulation determines resistance to chemotherapy through increased mitochondrial cholesterol accumulation [95]. Similar behavior was observed in cholesterol-enriched mitochondria or liposomes and reversed by restoring mitochondrial membrane order or cholesterol extraction. Cholesterol inhibited the membrane-permeabilizing activity of tBID/BAX or BAX pre-oligomerized with octylglucoside in a dose-dependent manner. Similar to the effect found on BAX, cholesterol also decreased the permeabilizing activity of melittin, a widely studied antimicrobial peptide, which induces membrane permeabilization by forming lipid-containing toroidal pores rather than through the formation of protein channels [17]. These findings indicate that cholesterol-mediated decrease in membrane fluidity of the bilayer directly modulates BAX pro-apoptotic activity by reducing the capacity of BAX to insert into the lipid matrix of the membrane, underlying the anti-apoptotic role of mitochondrial cholesterol accumulation in cancer cells. Thus, mitochondrial cholesterol contributes to chemotherapy resistance in HCC by increasing membrane order and resistance of MOM to MOMP. As StARD1 regulates mitochondrial cholesterol trafficking, it is conceivable that this member of the StART family stands as a novel target to regulate cancer cell death and chemotherapy response.

## Cancer biology and therapeutics

**Fig. 3:**
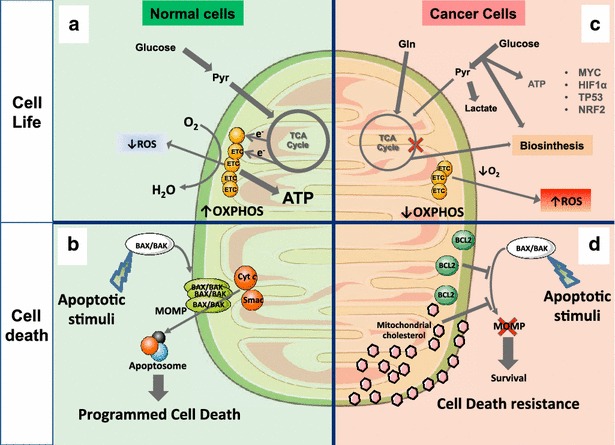
General summary of altered mitochondrial functions in cancer cell life and death. **a** In normal non-transformed cells glucose is mainly metabolized through the glycolysis pathway and the resulting pyruvate enters the mitochondrial TCA cycle, producing reduced equivalents that are fed into the ETC to generate ATP with high efficiency through the OXPHOS. Antioxidant defenses and coupled respiration through OXPHOS maintain low levels of mitochondrial ROS. **b** Normal cells are sensitive to apoptotic stimuli triggered by different stresses that finally converge in BAK/BAX activation and MOMP with subsequent release of cytochrome *c* into the cytosol, stimulating the formation of the apoptosome and apoptotic cell death. **c** In cancer cells gain-of-function of oncogenes and loss-of-function of tumor suppressor genes (such as MYC, HIF1α and TP53) results in altered metabolism, exemplified by the *Warburg effect*, characterized by high glucose consumption rates. Glucose is degraded through glycolysis to obtain biosynthetic intermediates and the resulting pyruvate is reduced to lactate to generate ATP. In this scenario, mitochondrial TCA is diverted to generate biosynthetic intermediates, which is accompanied by low OXPHOS activity and increased mitochondrial ROS production. NRF2 is upregulated in cancer cells to counteract ROS and permits cancer cells to withstand its deleterious effects. **d** Cancer cells, through the overexpression of anti-apoptotic proteins or inactivation of pro-apoptotic proteins, counteract the action of BAX/BAK and evade MOMP formation. Besides this effect, mitochondrial cholesterol loading shields mitochondrial membrane, impairing BAK/BAX oligomerization in MOM and subsequent MOMP formation and represents an additional mechanism of cell death resistance in tumor cells

As described in the previous sections, cancer cells exhibit critical metabolic transformations induced by mutations that result in cell cycle deregulation associated with enhanced cellular stress. Adaptation to this stress phenotype is required for cancer cells to survive and involves the participation of genes that regulate metabolism, bioenergetics, cell death and ROS detoxification (Fig. 3). In this context, small molecules that selectively kill cancer cells while sparing normal surrounding cells, are the desired approach for the treatment of cancer. To this aim, cancer therapeutics should target the differential features of cancer cells. Here, we briefly summarize the therapeutic strategies that involve mitochondria and their proposed mechanism of action to selectively target transformed cells.

### Therapeutics aimed at cancer metabolism and bioenergetics

Cells are addictive to glucose and glutamine and their limitation can cause cell death. This dependence is driven by the activation of MYC and HIF1-α [109] and consequently, targeting pathways regulating glucose/glutamine metabolism may be of relevance for cancer treatment. The specific GLS1 inhibitor* bis*-2-(5-phenylacetamido-1,3,4-thiadiazol-2-yl)ethyl sulfide (BPTES) inhibits proliferation of lymphoma cells but has no effect on neuroblastoma cells, which express GLS2 [214, 215], implying that the general GLS inhibitor 6-diazo-5-oxo-L-norleucine (DON) may exhibit broader antitumor effects [216, 217]. Inhibitors of glutamate dehydrogenase (GDH) are promising agents to target glutamine addiction of certain cancer cells. For instance, the green tea component epigallocatechin-3-gallate (EGCG), which inhibits GDH, has been shown to promote apoptosis in several cancers types, resulting in tumor growth inhibition, setting the basis for the exploration of its efficacy in phase II clinical trials [214, 218].

The inhibition of aerobic glycolysis in cancer cells is also of potential relevance. While inhibitors such as 2-deoxy-d-glucose and ionidamine, which targets early steps in the glycolysis pathway, exhibit severe toxic side effects [218], inhibition of distal steps in glycolysis are effective. Inhibition of lactate production by inactivation of lactate dehydrogenase (LDH) reduces tumorigenicity in several cancer models [219–221]. Additionally, the specific inhibitor of LDHA, FX11, reduces tumor progression in lymphoma in vivo [219]. In some cancers LDHB can replace LDHA, hence limiting the efficacy of the LDHA inhibitors [109, 222]. Inhibition of lactate export from cancer cells results in wide-reaching consequences, leading not only to lactate accumulation, alterations in glycolytic intermediates, reduction in glucose transport and ATP, NADP and GSH levels but also in mitochondrial damage and cell death [223], suggesting that inhibition of lactate transporter MCT1 is a suitable therapeutic approach. Moreover, the effect of small molecules that block the entry of pyruvate to the mitochondrial TCA cycle, such as dichloroacetate, a PDK1 inhibitor, may be of potential relevance [109, 218, 224].

Although targeting glycosis may be effective in a specific population of cancer cells exhibiting a highly glycolytic dependence, stem cancer cells that rely on OXPHOS might become resistant. Moreover, although mitochondrial oxidation under the Warburg effect is dramatically reduced, many cancer cells still have a central requirement on mitochondrial metabolism, strongly suggesting that OXPHOS inhibitors might represent an important target for drug-resistant cancers [121]. A key agent with potential relevance in inhibiting OXPHOS is metformin, one of the most prescribed drugs around the world for the treatment of type II diabetes [225–228]. Metformin is an indirect activator of AMP-activated Kinase (AMPK) through inhibition of mitochondrial complex I, resulting in the activation of the ATM/LKB1/AMPK axis. LKB1 is a well-characterized tumor suppressor in pancreatic, lung cancer and melanoma. AMPK activation inhibits the mTOR pathway and this effect accounts for the potential antineoplastic effects of metformin in breast and renal tumors. Moreover, metformin reduces glycolysis and increases mitochondrial respiration in tumors, and these events are associated with growth arrest [229]. In addition, metformin exhibits antiangiogenic effects, which contribute to its antineoplastic properties [230]. Other compounds with mild OXPHOS inhibition such as tamoxifen, which also inhibits complex I, resveratrol, which antagonizes complex III, and the complex V inhibitor 3,3-diindolylmethane have potential in cancer treatment. VLX600 is a novel compound targeting OXPHOS that inhibits tumor growth of colon carcinoma cells, thus exhibiting potential application in clinical trials [231]. Besides, a number of emerging mitochondrial inhibitors successfully used in experimental studies could be effective against cancer cells and might synergize with chemotherapeutics [149, 232, 233].

### Therapeutics targeting cancer cell death

Given that BCL-2 is overexpressed in many tumors, most strategies to engage apoptosis pathways are based in the blockade of anti-apoptotic members of the BCL-2 family. BCL-2 inhibitors have been developed based on the structure of BH3-binding groove of BCL-xL [234], leading to the development of the prototypic BH3 mimetic that displays sub-nanomolar affinity for BCL-xL and a binding profile similar to the BH3-only protein BAD. The BH3 mimetic ABT-737 and the more soluble analogue ABT-263 bind BCL-xL, BCL-2 but not MCL1 and both show antitumor activities either as single agents or in combination [235]. However, the clinical applicability of these BH3 mimetics is limited due to severe thrombocytopenia mediated by platelet apoptosis [236, 237]. ABT-199, a novel BH3 mimetic developed from the structure of ABT-263 [238], is effective in chronic lymphocyte leukemia. A potential side effect of ABT-199 is the induction of tumor lysis syndrome [239], which can be controled by step-wise dose escalation. Although the therapeutic results with BH3 mimetics are promising, resistance is a potential drawback. For instance, BCL-2 mutations that abrogated binding of BH3 mimetics mediate resistance of ABT-199 in experimental lymphoma models [240]. As BCL-2 inhibitors do not target MCL-1, a key anti-apoptotic BCL-2 member, the efficiency of BH3 mimetics may be limited, particularly in the treatment of solid tumors [241–243]. Hence, the combination of specific MCL1 inhibitors [244, 245] with BH3 mimetics is a promising therapeutic approach to overcome chemotherapy resistance. Moreover, as MCL-1 plays a key role in mitochondrial physiology and autophagy, targeting MCL-1 may cause undesirable side effects [246, 247]. For instance, the toxicity of pan-BCL-2 inhibitors, such as Gossypol or Obatoclax, which inhibit BCL-2 and MCL1, prevented the evaluation of their efficacy in clinical settings [243]. These findings suggest that MCL1 inhibition should be fine-tuned and that the relative contribution of BCL-2-family components to the apoptosis resistance of cancer cells should be carefully evaluated through the “BH3 profiling” to determine the therapeutic window [248, 249]. In addition, incomplete cell death caused by triggers of mitochondrial apoptosis can promote genomic instability and mutagenesis derived from the incomplete MOMP and caspase-dependent DNA cleavage, contributing to tumor relapse and the acquisition of drug resistance [212, 213]. Based on the ability of TP53 to induce apoptosis, mitochondrial targeted TP53 fusion proteins have been developed to induce intrinsic apoptosis in cancer cells, which may be of relevance in adjuvant therapy for cancer treatment [201, 250]. Overall, targeting or sensitizing cancer cells to apoptosis is a promising strategy currently under development, which may lead to personalized medicine through specific tumor-profiling and fine-tuning dosage and therapy combinations.

### Therapeutics targeting cancer cell ROS sensitivity

Despite generation of higher ROS levels cancer cells are more sensitive to intracellular ROS induction than untransformed cancer cells. Many cancer chemotherapeutic agents, including taxanes, vinca alkaloids, platinum coordination complexes, paclitaxel and elesclomol are currently used to induce high levels of ROS to kill cancer cells [251]. The ultimate effect of these molecules is determined by the intrinsic antioxidant capacity of cancer cells as the cytotoxic potential of these agents is lost upon antioxidant co-treatment [252–254].

A key mechanism to counteract the generation of ROS by chemotherapeutic agents is the regulation of GSH homeostasis [137, 182]. Several small molecules, which modulate ROS, such as β-phenethyl isothiocyanate (PEITC), buthionine sulphoximine (BSO), curcumin or CDDO derivatives, have potential therapeutic effects for the treatment of cancer by promoting mGSH deletion and subsequent ROS generation specifically in cancer cells [255–258]. BSO, an inhibitor of glutamate-cysteine ligase, which is the rate-limiting enzyme in GSH biosynthesis [259] is the only clinically used drug to suppress the novo GSH synthesis. The simultaneous administration of BSO and the thioredoxin inhibitor auranofin induce ROS and clonogenic killing in carcinoma cells [260]. Sulfasalazine, which inhibits cystine uptake via XcL carrier, limits cysteine availability impairing GSH biosynthesis, which leads to reduced growth and viability of cancer cells in vitro and in vivo [261, 262]. In addition, specific mGSH depletion has also been associated with apoptosis induced by chemotherapeutic drugs. For example, the triterpenoid methyl CDDO derivative (CDDO-Me), induces cytotoxicity in chemotherapy-resistant myeloid leukemia cells and this event is associated with selective depletion of mGSH, resulting in increased ROS generation [263, 264]. Moreover, PEITC depletes mGSH and consequently increases ROS and nitric oxide, contributing to inhibition of the mitochondrial complex I, suppression of mitochondrial respiration, and subsequent cytotoxicity of leukemia cells [265]. Using a cell-based small-molecule screening and quantitative proteomics, piperlongumine has emerged as a cytotoxic agent that triggers apoptosis and necrosis in leukemia cells [266]. Interestingly, piperlongumine induces ROS generation and cell death in transformed cells but not primary normal cells [267]. Piperlongumine also decreases GSH and increases GSSG levels in cancer cells without effects in nontransformed cells, and these effects parallel the ability of piperlongumine to alter mitochondrial morphology and function. Consequently, co-treatment with piperlongumine and NAC prevented piperlongumine-mediated GSH depletion and cell death in cancer cells. These findings support the concept that cancer cells have high levels of ROS, and hence, have a strong reliance on the ROS stress-response pathway driven by NRF2.

At present, radiotherapy is widely used in various types of cancer treatments, and the therapeutic effect is mainly determined by ROS generation. The induction of water radiolysis occurs in seconds after ionizing radiation, lasts several hours after exposure and enhances ROS generation and oxidative stress [268, 269]. Some studies suggested that antioxidant supplementation could sensitize cancer cells to chemo- or radio-therapy and reduce their side effects by protecting the normal cells [270]. However, other studies indicated that antioxidants may also protect cancer cells against these therapies [252, 271, 272]. Therefore, the safety and efficacy of the combined treatment of antioxidants with radiotherapy or ROS-inducing chemotherapy remain controversial.

### Strategies targeting the mevalonate pathway and cholesterol synthesis in cancer

As described above, cancer cells exhibit alterations in the regulation of cholesterol homeostasis and *de novo* synthesis in the mevalonate pathway. Despite that the main therapeutic benefit of statins is the prevention of cardiovascular diseases and heart attacks, the use of statins has been associated with lower incidence of colorectal carcinoma, melanoma, prostate cancer and HCC, although the benefit of statins in other types of cancer has been disappointing [273]. While statins inhibit cholesterol synthesis, they also affect other intermediates of the mevalonate pathway, including isoprenoids, and therefore the beneficial effects of statins in cancer may be independent of cholesterol synthesis. For instance, statins inhibit the activation of the proteasome pathway, contributing to the maintenance of proteins that block cell cycle. Through cholesterol downregulation, statins regulate the function of Hedgehog, a signaling pathway involved in carcinogenesis [273]. Besides these wide-reaching effects of statins, their benefit in cancer treatment is limited due to the complex regulation of HMGCoAR and the metabolites generated in the mevalonate pathway. Reduction of isoprenoid and cholesterol levels in cancer by chronic treatment with statins leads to upregulation of HMG-CoAR levels and eventually development of resistance [274]. In vitro mechanistic studies of statins used significantly higher concentrations than those that were therapeutically achievable in phase I trials. Dose-limiting toxicities, including gastrointestinal side effects, myelotoxicity, myalgias, elevation of creatine phosphokinase and hepatotoxicity, precluded further dose increase in clinical trials [275]. Inhibition of SS has attracted much interest as a pharmacological target as it implies the inhibition of cholesterol synthesis without depressing isoprenoid levels. For instance, lapaquistat (TAK-475, Takeda), a SS inhibitor, progressed to phase III clinical trials, although its outcome in cancer remains to be established due to hepatotoxic effects at high dosing [276]. As mentioned before, prenylation is a key postranslational mechanism of targeted proteins, and many prenylated proteins are involved in various aspects of carcinogenesis, including cellular proliferation, apoptosis, angiogenesis and metastasis. Farnesylation is catalyzed by farnesyltransferase (FTase) and geranylgeranylation by geranylgeranyltransferase, GGTase. Given the role of protein prenylation in carcinogenesis, FTase inhibitors (FTIs) and GGTase inhibitors (GGTIs) have been developed for cancer treatment. GGTI–FTI combinations synergistically inhibit proliferation of multiple myeloma cell lines and primary cells, and induce apoptosis. Interestingly, dual prenylation inhibitors (DPIs) that block both FTase and GGTase enzymatic activities have been shown to induce apoptosis in PSN-1 pancreatic tumor cells by blocking *K*-Ras prenylation compared to either FTI or GGTI agents alone [277]. H and N-Ras prenynation is effectively inhibited by FTIs and only partially by GGTIs, whereas K-Ras prenylation requires both FTIs and GGTIs inhibition [278]. Thus, combined inhibition of geranylgeranylation and farnesylation can overcome the resistance conferred by cross-prenylation, thus potentiating the activity of either FTIs or GGTIs alone. Finally, targeting the specific targeting of cholesterol to mitochondria may be an additional approach of potential benefit in cancer treatment by modulating cell death and chemotherapy resistance. This specific field is currently under investigation to identify potential specific inhibitors of StARD1 and MLN64 to sensitize cancer cells to cell death triggers and chemoterapeutic agents.

## Conclusions and future approaches

Cancer cells undergo an array of genetic and epigenetic modifications that lead to a phenotype characterized by high proliferation, death resistance, rapid growth and invasiveness. Mitochondria play an essential role in metabolism, bioenergetics and cell death regulation and consequently oncogenic modifications characteristic of many cancer types mediate the array of metabolic alterations of cancer cells by impairing key mitochondrial functions. This continuum evolving process in the adquisition of a highly proliferative phenotype requires the selection of cells with decreased mitochondrial oxidation of fuels, relying on the oxidation of glucose for ATP generation, resulting secondarily in the engagement of the pentose phosphate pathway as a source of reducing equivalents needed for anabolism and antioxidant defense. These metabolic alterations are accompanied by the involvement of mitochondria in biosynthetic pathways to support continuous growth, while reducing the deleterious effects of high-rate production of ROS, a characteristic feature of cancer cells. Furthermore, mitochondria undergo changes in membrane dynamics, exemplified by the decrease in membrane fluidity to protect cancer cells against the induction of programmed cell death triggered by the immune system or by metabolic or xenobiotic stresses. A key player in this event is the accumulation of cholesterol in mitochondria of cancer cells, which increases the threshold for MOMP by restructuring mitochondrial membrane bilayers. Besides this function, mitochondrial cholesterol accumulation may indirectly contribute to the metabolic changes of cancer cells by impairing mitochondria function and activation of survival programs turned on by HIF1α activation. Given these functions of mitochondrial cholesterol, preventing or reversing this process may be of relevance in cancer cell biology to shift the balance towards increased apoptosis susceptibility and sensitization to chemotherapy. In addition, oncogenes, transcription factors (e.g. MYC, HIF1α, NRF2) and inactivation of tumor suppressors, such as TP53, allow invasiveness and chemoresistance, in part, by regulating mitochondrial function and metabolism as well as by controling the outcome of ROS generation. Metabolic stress, immune surveillance and chemotherapy act as a selective pressure that allows only the survival of cells with specific features, driving cancer cells towards a highly glycolytic, apoptosis-incompetent and invasive phenotype. Given the complexity in the metabolic alterations of cancer cells mediated largely through alterations in mitochondrial function, further research is required to identify more efficient strategies for cancer treatment involving the use of small molecules targeting mitochondrial metabolism.

## Abbreviations

ROS: reactive oxygen species
OXPHOS: oxidative phosphorylation
HMGCoAR: 3-hydroxy-3-methyl-glutaryl-CoA reductase
HCC: hepatocellular carcinoma
TCGA: cancer genome atlas
mtDNA: mitochondrial DNA
MIM: mitochondrial inner membrane
MOM: mitochondrial outer membrane
ALR: augmenter of liver regeneration
ETC: electron transport chain
Δψ_m_: mitochondrial transmembrane potential
TCA: tricarboxylic acid cycle
ACLY: ATP citrate lyase
mGSH: mitochondrial glutathione
H_2_O_2_: hydrogen peroxide
SOD: superoxide dismutases
2-OG: 2-oxoglutrarate
GPX: glutathione peroxidase
mGSSG: mitochondrial oxidized glutathione
Prx/Trx: peroxiredoxin/thioredoxin
MOMP: mitochondrial outer membrane permeabilization
IMS: mitochondrial intermembrane space
BOK: BCL-2 ovarian killer
MPT: mitochondrial permeability transition
VDAC: voltage-dependent anion channel
ANT: adenine nucleotide translocase
TSPO: translocator protein
RIPK1: receptor-interacting serine/threonine-protein kinase 1
MLKL: mixed lineage kinase domain-like protein
IPP: isopentenyl pyrophosphate
DMAPP: dimethyl allyl pyrophosphate
GPP: geranyl pyrophosphate
FFP: farnesyl pyrophosphate
GGPP: geranyl geranyl pyrophosphate
SS: squalene synthase
ER: endoplasmic reticulum
ABCA1: ATP binding cassette transporter A1
StARD1: steroidogenic acute regulatory domain 1
PET: positron emission tomography
PDK1: pyruvate dehydrogenase kinase
ARK5: AMPK-related protein kinase 5
PGC1α: peroxisome proliferator-activated receptor gamma coactivator 1 alpha
GLS2: glutaminase 2
HIF1α: hypoxia-inducible factor 1 alpha
PHD: HIF-proly-hydroxylase
pVHL: Von Hippel-Landau ubiquitin ligase
LDHA: lactate dehydrogenase A
MCT4: monocarboxylate transporter 4
COX4-2: cytochrome c oxidase subunit 4–2
BNIP3: BCL-2/adenovirus E1B 19-kDa interacting protein 3
2-OG: 2-oxoglutarate
FIH: factor inhibiting HIF1
SDH: succinate dehydrogenase
IDH: isocitrate dehydrogenase
NAC: *N*-acetyl-l-cysteine
NRF2: NF-E2-related factor
KEAP1: Kelch-like ECH-associated protein 1
BHA: butylated hydroxyanisole
CDDO: 2-cyano-3,12-dioxooleana-1,9-diene-28-oic acid
FH: fumarate hydratase
2-OGC: 2-oxoglutarate carrier
DRP1: dynamin-related protein 1
BPTES: *bis*-2-(5-phenylacetamido-1,3,4-thiadiazol-2-yl)ethyl sulfide
DON: 6-diazo-5-oxo-L-norleucine
GDH: glutamate dehydrogenase
EGCG: epigallocatechin-3-gallate
LDH: lactate dehydrogenase
AMPK: AMP-activated Kinase
PEITC: β-phenethyl isothiocyanate
BSO: buthionine sulfoximine
FTase: farnesyltransferase
FTI: farnesyltransferase inhibitors
GGTI: geranyl geranyl transferase inhibitor
